# A cross‐sectional study of early mobility practice in intensive care units in Sarawak Hospitals, Malaysia

**DOI:** 10.1002/nop2.619

**Published:** 2020-10-16

**Authors:** Siew Yieng Yong, Sidiah Siop, Wee Meng Kho

**Affiliations:** ^1^ Advanced Diploma in Intensive Care Nursing Ministry of Health Malaysia Training Institution Kuching Malaysia; ^2^ Nursing Department Faculty of Medicine and Health Sciences Universiti Malaysia Sarawak (UNIMAS) Kuching Malaysia; ^3^ Internal Medicine and Dermatology Timberland Medical Centre Kuching Malaysia

**Keywords:** adherence, early mobility, prevalence

## Abstract

**Aims:**

To determine the prevalence, characteristics of EM activities, the relationship between level of activity and mode of ventilation and adherence rate of EM protocol.

**Background:**

Mobilizing ICU patients remains a challenge, despite its safety, feasibility and positive short‐term outcomes.

**Design:**

A cross‐sectional point prevalence study.

**Methods:**

All patients who were eligible and admitted to the adult ICUs during March 2018 were recruited. Data were analysed by using the Statistical Package for Social Sciences version 24 for Windows.

**Results:**

The prevalence of EM practice was 65.6%. The most frequently reported avoidable and unavoidable factors inhibit mobility were deep sedation and vasopressor infusion, respectively. Level II of activity was the most common level of activity performed in ICU patients. The invasive ventilated patient had 12.53 the odds to stay in bed as compared to non‐invasive ventilated patient. An average adherence rate of EM protocol was 52.5%.

## 1 INTRODUCTION

Conventionally, hospital mortality or short‐term physiologic end points were emphasized as the outcome of patients requiring mechanical ventilation (Combes et al., 2003; Zilberberg & Epstein, 1998). Nevertheless, functional status of ICU patients attracts clinicians’ attention following a high proportion of ICU survivors suffer from significant physical disabilities, secondary to neuromuscular weakness from critical illness, prolonged bed rest and immobility (Bailey et al., 2007; Burtin et al., 2009; Kress, 2009; Morris et al., 2008; Morris & Herridge, 2007; Perme & Chandrashekar, 2009). Early mobility (EM) of ICU patient has been reported as safe, feasible and beneficial on ICU patients and by maximizing independent function (Burtin et al., 2009; Hodgson et al., 2013; Morris et al., 2008; Schweickert & Kress, 2011; Schweickert et al., 2009; Tipping et al., 2016).

In Malaysia, Malaysian Society of Intensive Care (MSIC) audited the adherence rate of EM protocol among the hospitals each year. The adherence rate of EM protocol among 43 government ICUs varies from 28% to 100%, with a reported average of 74.5% in the year 2016 (Tai et al., 2017). Nevertheless, the prevalence of EM practice remains unknown as there is no prior study. This study aimed to determine the prevalence of EM practice, characteristics of the mobility activities for ICU patients, the relationship between level of activity and mode of ventilation and adherence rate of EM protocol in Sarawak ICUs.

## 2 BACKGROUND

**Figure 1:**
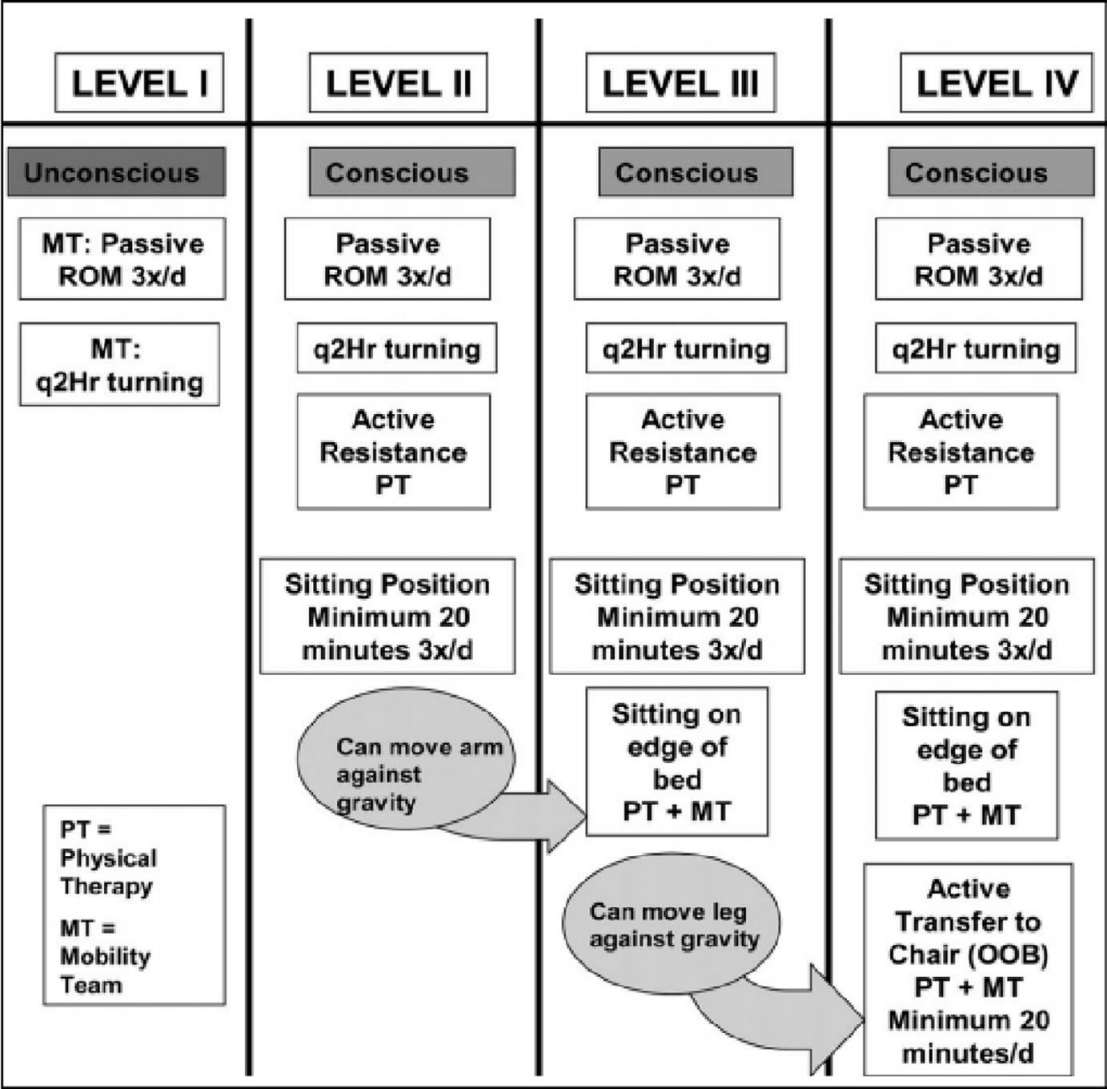
A step‐wise programme of mobility (level I to level IV) in Ministry of Health Malaysia Early Mobility protocol (Management Protocol in ICU, 2012) available online at www.msic.org.my/download/Management ProtocolsInICU.pdf

In this study, EM is defined as physical activity to be initiated to a patient in 24 to 48 hr after ICU admission in the absence of contraindication(s) according to EM protocol for MOH Malaysia ICUs (Tong et al., 2014). MOH Malaysia ICUs adopted Morris et al. (2008)’s framework of mobility activity in 2013. The framework consists of a step‐wise programme of mobility (level I to level IV) based on the consciousness level and functional ability of patients as shown in Figure 1.

### 2.1 Prevalence of EM practice

Ambulating mechanically ventilated patient is not seen to be a common practice (Bakhru et al., 2015; Barber et al., 2014) although growing bodies of study on EM practice (Burtin et al., 2009; Green et al., 2018; Perme & Chandrashekar, 2009; Pohlman et al., 2010) had reported the feasibility and benefits of EM following Bailey et al. (2007)’s study. Jolley et al. (2014) reported that the provision of EM had not been widely adopted despite the feasibility and benefits of EM.

Harrold et al. (2015) conducted a four‐week prospective observational cohort study at ten ICUs in Australia and nine ICUs in Scotland to compare EM practice between these two countries. The results of the study revealed that 60.2% of the 347 patients at ICUs in Australia and 40.1% of the 167 patients at ICUs in Scotland were mobilized during their ICU stay (*p* < .001). Similarly, Leditschke et al. (2012) also reported that only 54% of patient‐days in Australia involved patients with EM, and avoidable factors were identified in 47% of cases when patients were not mobilized.

In Malaysia, conventional approach of in‐bed mobility for ICU patients is still mainly being practised in ICUs. Of late, Leong et al. (2017) conducted a descriptive cross‐sectional survey to assess EM practice among 186 nurses in ICUs at University Malaya Medical Centre in Malaysia. Results of the study revealed that in‐bed mobility was reported by almost all of the nurses (*n* = 131, 99.2%) in the study, and almost half of the respondents (*n* = 63, 47.7%) solely performed PROM to mechanically ventilated patients.

### 2.2 Adherence of clinical practice guidelines

Otterman et al. (2012) conducted a national survey in the Netherlands using a web‐based questionnaire to assess the adherence of current clinical practice guidelines for patients with stroke (CPGPS). Results of the survey revealed a large gap between current practice and the evidence‐based recommendations in the Dutch CPGPS. The mean amount of exercise therapy provided by PTs in Dutch Acute Hospital Stroke Units was approximately half (22 min/day) of the recommended time of 40 min/day. Similarly, a survey conducted in the United States reported that at least 30%–40% of patients did not receive nursing care as according to current scientific evidence (Grol & Wensing, 2004).

According to the MSIC 2015 report (Tong et al., 2016), there was a considerable variation in EM protocol adherence rate reported among 43 Ministry of Health ICUs in the year 2015. The EM protocol adherence rate ranged from 32.9% to 100%, with an average of 71.5% (Tong et al., 2016). As for Sarawak, the adherence rate for Sarawak General Hospital (SGH), Sibu Hospital, Bintulu Hospital and Miri Hospital was 33.4%, 97.5%, 69.1% and 84%, respectively. The overall adherence rate had slightly improved by 3% to 74.5% as published in the MSIC 2016 report (Tai et al., 2017). Nevertheless, the average EM protocol adherence rate of the four hospitals in Sarawak declined approximately 10% (Tai et al., 2017).

#### 2.2.1 Research questions

The following research questions are addressed:

1. What is the prevalence of EM practice in the Sarawak ICUs?
2. What are the characteristics of EM activities in the Sarawak ICUs?
3. What is the relationship between level of activity and mode of ventilation?
4. What is the adherence rate of EM protocol in the Sarawak ICUs?

## 3 METHODS

### 3.1 Design

A 4‐week cross‐sectional point prevalence study.

### 3.2 Study period

A four‐week cross‐sectional point prevalence study was carried out from 1st to 31st March 2018 to determine the EM practice in Intensive Care Units in Sarawak Hospitals.

### 3.3 Study location

This study was carried out at five multidiscipline adult ICUs in Sarawak which adopted the Early Mobility protocol, namely SGH, Sarikei Hospital, Sibu Hospital, Bintulu Hospital and Miri Hospital with a capacity of 17, 6, 17, 6 and 8 beds, respectively. SGH is the largest, state referral hospital in Sarawak. SGH and Sibu hospital are categorized as level III ICU in the MOH and are managed by intensivists. According to the Anaesthesiology and Intensive Care Service Operational Policy (Anaesthesiology Intensive Care Services Operational, 2013), level III ICU shall be a combined medical and surgical unit, with the number of beds ranging from 16–35, facilities to support multiple organ failure, and is headed by an intensivist or an anaesthetist with special interest in intensive care. Sarikei Hospital, Bintulu Hospital and Miri Hospital are categorized as level II ICU in the MOH. Level II ICU is defined as ICU located in district hospitals with the number of beds ranging from 6–10, presence of anaesthetists who are capable of providing intensive care and mechanical ventilation to patients (Anaesthesiology Intensive Care Services Operational, 2013).

### 3.4 Study sample

A patient‐day is counted for each day that a patient is in the ICU (Leditschke et al., 2012) during the four weeks of study days. Universal sampling was used in this study as all the patients who were eligible during the study period were recruited in this study.

### 3.5 Study population

The eligibility criteria for recruitment of participants in the study were in accordance with the EM protocol for MOH Malaysia ICUs (Lee, 2012):

#### 3.5.1 Inclusion criteria

1. Myocardial stability with systolic blood pressure >90 mmHg, heart rate <120 beats/min, no evidence of acute myocardial ischaemia in the last 24 hr and absence of dysrhythmia requiring new anti‐dysrhythmic agents in the last 24 hr.
2. Oxygenation adequacy (O) was defined as patients who met the parameters of FiO_2_ ≤ 0.6, PEEP ≤ 10 cm H_2_O, SPO_2_ > 90% and respiratory rate < 35 per minute.
3. Without use of two or more vasopressor(s)/inotrope(s) infusion; and an increase of any vasopressor(s)/inotrope(s) in the last 24 hr.

#### 3.5.2 Exclusion criteria

1. Absolute contraindications, such as susceptible/known dissecting aneurysm; cerebral protection, high intracranial pressure and active intracranial bleed; and unstable spinal injury/pelvic fracture.
2. On continuous renal replacement therapy (CRRT)
3. Restless

### 3.6 Research variables

#### 3.6.1 Independent variables

The independent study variables used in this study were as follows:

1. Number of eligible patients involved in EM
2. Mode of ventilation
3. Total number of activities participated by patients according to their level of activity

#### 3.6.2 Dependent variables

The dependent study variables or the major outcomes of this study were as follows:

1. Prevalence of EM practice
2. Characteristics of EM activities
3. Adherence rate of EM protocol

### 3.7 Tools

**Figure 2:**
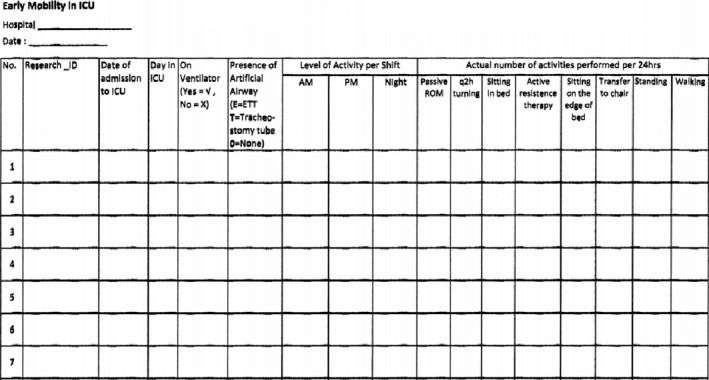
Self‐developed Early Mobility form

A specific form of EM (Figure 2) was developed by the investigator in order to assess the prevalence and determine the adherence rate of EM protocol in ICUs. The Strengthening the Reporting of Observational Studies in Epidemiology (STROBE) checklist was used as guideline in reporting this study (see Supplementary File S1).

### 3.8 Data collection

A research coordinator was appointed from each hospital to assist in data collection. For each day during the study period, the research coordinators would identify eligible patients for EM by checking on the MOH EM assessment forms which were recorded by the bedside nurses. For each eligible patient, the level of activity was identified from the MOH EM assessment form. The total number of EM activities performed over 24‐hr period (8 a.m. to 8 a.m. the next day) was counted from the MOH EM activity form and was recorded into the appropriate columns in the specific EM form. Mode of ventilation applied on each patient was recorded. The reason(s) patient who was not eligible to EM was justified in the specific EM form. For standardization of data collection, research coordinators were trained to extract data from the MOH EM assessment and activity forms and record it into the specific EM form accordingly.

### 3.9 Statistical analysis

Jolley et al. (2016) defined the prevalence of mobility activity as the proportion of patient‐days with mobility event during the study period. A patient‐day is counted for each day that a patient is in the ICU during the study days (Leditschke et al., 2012). In this study, the prevalence of EM practice was measured as the proportion of patient‐days with EM during the four‐week study period in the ICUs.

$$\text{Prevalence}=\frac{\text{Total}\;\text{number}\;\text{of}\;\text{patient}\;‐\;\text{days}\;\text{with}\;\text{EM}}{\text{Total}\;\text{number}\;\text{of}\;\text{patient}\;‐\;\text{days}\;\text{during}\;4\;\text{weeks}\;\text{of}\;\text{study}\;\text{period}}$$

The calculation of adherence rate in this study adopted the formula from Tong et al. (2016) as the percentage of actual number of activities performed against the required number of activities to be performed by all eligible patients in the ICUs. The total number of activities required for each level of activity is according to the MOH EM protocol (Lee, 2012).

$$\text{Adherence}\;\text{rate}=\frac{\text{Total}\;\text{actual}\;\text{number}\;\text{of}\;\text{activities}\;\text{performed}}{\text{Total}\;\text{number}\;\text{of}\;\text{activities}\;\text{required}\;\text{to}\;\text{be}\;\text{performed}}$$

Descriptive statistics were performed to determine the characteristics of the mobility activities for ICU patients during the study period. Pearson chi‐square test in 2 × 2 contingency tables was performed to determine the relationship between the mode of ventilation and the level of activity, with significant level, *α* = 0.05. For the two categorical variables, characteristics of EM activities were categorized into in‐bed and out‐of‐bed activities, which indicated activity of levels I and II and activity of level III and IV, respectively (Tai et al., 2017). Mode of ventilation was categorized into invasive (use of ETT and tracheostomy tube) and non‐invasive (use of non‐invasive positive pressure ventilation and venture mask/nasal prong) ventilation. Odds ratio was calculated to measure the association between the two variables. All data collected were analysed by using the Statistical Package for Social Sciences (SPSS) version 24 for Windows.

### 3.10 Ethics approval and consent to participate

The study was conducted upon obtaining approval from the National Medical Research and Ethics Committee and the Institutional Research Ethics Committee. Permission to conduct the study was also obtained from the study sites.

## 4 RESULTS

### 4.1 Study day characteristics

During the four‐week of study, a total of 1,344 patient‐days were collected for EM practice in ICUs in Sarawak Hospitals after excluding seven missing data. A patient‐day is a patient's presence in ICU each day during the study period. Out of 1,344 patient‐days, there were 882 patient‐days involved EM in ICUs. A total of 268 patients contributed to the 882 patient‐days data. The mean of ICU stay for patient in this study was 3.29 days. Majority of the patients received mechanical ventilation via an endotracheal tube, ETT (*n* = 583, 66.1%). The frequency and percentage distribution of study‐days characteristics are presented in Table 1.

**Table 1:** Frequency and percentage distribution of patient‐days according to hospitals, study day characteristics, prevalence of EM practice in ICUs, level of activity and initiated level of activity (*n* = 882 patient‐days)

	Frequency (*n*)	Percentage (%)
Hospital		
Sarawak General Hospital	315	35.7
Sarikei Hospital	45	5.1
Sibu Hospital	329	37.3
Bintulu Hospital	68	7.7
Miri Hospital	125	14.2
Study day characteristics		
Endotracheal tube	583	66.1
Tracheostomy	100	11.3
Non‐invasive positive pressure ventilation	65	7.4
Nasal prong/venturi mask	134	15.2
Prevalence of EM practice		
Involved EM	882	65.6
Without EM	462	34.4
Age < 18 years old	69	5.1
Less than 24 hr	228	17.0
Barriers to mobility	165	12.3
Unstable spinal injury	9	5.5
Vasopressor	55	33.3
Oxygenation inadequacy	22	13.3
On CRRT	9	5.5
Restless	16	9.7
Cerebral protection	1	0.6
Myocardial instability	21	12.7
Deep sedation	32	19.4
Level of activity		
I	371	42.0
II	410	46.5
III	51	5.8
IV	50	5.7
Initiated level of activity		
I	882	65.6
II	511	38.0
III	101	7.5
IV	50	3.7
Adherence rate of EM protocol		
Sarawak General Hospital		48.3
Sarikei Hospital		54.1
Sibu Hospital		46.9
Bintulu Hospital		74.2
Miri Hospital		65.5

Abbreviation: CRRT, continuous renal replacement therapy.

### 4.2 ICU characteristics

For the 882 patient‐days of study, Sibu hospital contributed the most frequent patient‐days (*n* = 329, 37.3%). The frequency and percentage distribution of patient‐days according to hospitals in this study are presented in Table 1.

### 4.3 Prevalence of EM practice in the ICUs

The prevalence of EM practice in the government ICUs of Sarawak was 65.6% (*n* = 882 patient‐days out of total of 1,344 patient‐days). Barriers to mobility (*n* = 165, 12.3%) contributed the highest percentage of patient‐days without EM activity, with potentially avoidable factors constituted 28.8% patient‐days which included deep sedation and restless.

For the unavoidable factors that hinder mobility, patients on vasopressor (*n* = 55, 33.3%) was the most frequently reported factor. The prevalence of EM practice in the ICUs is presented in Table 1.

#### 4.3.1 Characteristics of the mobility activities

In this study, level II activity recorded as the highest frequency, which consisted of 46.5% (*n* = 410 patient‐days) as shown in Table 1.

#### 4.3.2 Initiated level of activity

The prevalence of EM practice declined dramatically if the initiated level of activity ascending from level I–IV (Table 1).

### 4.4 Relationship between mode of ventilation and level of activity

Overall, level II activity was the most common level of activity performed in both invasively and non‐invasively ventilated patients. Nonetheless, level I activity was the most frequently performed activity in invasively ventilated patients (40.0%, *n* = 353 patient‐days). The frequency and percentage distribution of level of activity for invasive and non‐invasive ventilated patients in ICU are presented in Table 2. Result of Pearson chi‐square test showed there was a significant relationship between mode of ventilation and level of activity (*p* < .001). The invasive ventilated patient had 12.53 the odds to stay in bed as compared to non‐invasive ventilated patient. The relationship between mode of ventilation and level of activity is presented in Table 3.

**Table 2:** Frequency and percentage distribution of level of activity for invasive and non‐invasive ventilated patients in ICUs (*n* = 882 patient‐days)

Level of activity	Invasive		Non‐invasive	
	Frequency (*n*)	Percentage (%)	Frequency (*n*)	Percentage (%)
I	353	40.0	18	2.0
II	299	33.9	111	12.6
III	15	1.7	36	4.1
IV	16	1.8	34	3.9

**Table 3:** The relationship between mode of ventilation with level of activity (*n* = 882 patient‐days)

Level of activity	Mode of ventilation				*χ* ^2^ statistic^a^ (*df*)	*p* value^a^
	Endotracheal tube *n* (%)	Tracheostomy *n* (%)	NIPPV *n* (%)	Nasal Prong/Venturi Mask *n* (%)		
Level I	337 (38.2%)	16 (1.8%)	6 (0.7%)	12 (1.4%)	278.30 (9)	.000
Level II	232 (26.3%)	60 (6.8%)	38 (4.3%)	80 (9.1%)		
Level III	8 (0.9%)	16 (1.8%)	10 (1.1%)	17 (1.9%)		
Level IV	6 (0.7%)	8 (0.9%)	9 (1.0%)	27 (3.1%)		

Abbreviation: NIPPV, non‐invasive positive pressure ventilation.

^a^ Pearson Chi‐square.

### 4.5 Adherence rate of EM protocol

A total of five hospitals in this study had contributed to the 882 patient‐days data with an average adherence rate of 52.5% to EM protocol. There was a difference of 27.3% adherence rate between the highest (74.2%) reported in Bintulu Hospital and the lowest (46.9%) reported in Sibu Hospital. The EM protocol adherence rate of the five hospitals in Sarawak is shown in Table 1.

## 5 DISCUSSION

### 5.1 Prevalence of EM practice

The prevalence of EM practice in five government ICUs in Sarawak was 65.6% of patient‐days during a four‐week study in March 2018. A relatively higher prevalence was reported in this study as compared the existing studies conducted in Australia, Scotland and the United States (Harrold et al., 2015; Jolley et al., 2016; Leditschke et al., 2012), with the prevalence around 50%, 40% and 30%, respectively. Notably, the prevalence of EM in ICUs in existing literature (Harrold et al., 2015; Leditschke et al., 2012; Leong et al., 2017) showed discrepancies between the types and level initiated of mobility activity participated by ICU patients.

Notably, PROM and two hourly turning were excluded as part of EM activity in some studies (Harrold et al., 2015; Leditschke et al., 2012). Furthermore, the lowest level activity of sitting at the edge of bed, which is equivalent to level III activity in this study, was applied in Harrold's study (Harrold et al., 2015). A relatively lower prevalence of 7.5% (101 patient‐days) was reported in this study if sitting at the edge of bed (level III) was initiated for EM as compared to 60.2% and 40.1% reported in Australia and Scotland studies, respectively (Harrold et al., 2015).

#### 5.1.1 Characteristic of mobility activities

There was a remarkable decrease in the prevalence when the level of EM activity escalated from the lowest (level I) to the highest (level IV) in this study. Levels I and II activities in the EM protocol were reported as frequently participated by the ICU patients in Sarawak ICUs. Malaysia appears to be more conservative in mobilizing critically ill ICU patients as compared to the studies in existing literature (Harrold et al., 2015; Leditschke et al., 2012).

The findings of this study indicated that out‐of‐bed activities were not routinely practised in government ICUs in Sarawak. This finding was consistent with our counterparts in Peninsular Malaysia (Leong et al., 2017). Similarly, a 1‐day point prevalence study of mobility of mechanically ventilated patients in 116 ICUs across Germany revealed that approximately three‐quarter of patients (*n* = 185, 24%) were not mobilized out of bed; standing and higher level mobility were rarely occurring in ICUs. The reason could be that ICU patients who required mechanical ventilation were often perceived as “too sick” in their early phase of illness (Bailey et al., 2007; Schweickert & Kress, 2011), thus required deep sedation and bed rest (Castro et al., 2015; Leditschke et al., 2012). Recent evidence has suggested that mobilization is safe for patients undergoing CRRT (Brownback et al., 2014; Wang et al., 2014). Nevertheless, patients receiving CRRT in ICUs Sarawak have not been candidates for mobilization and have remained on strict bed rest.

The culture of keeping the ICU patients completely rest in bed impedes the implementation of EM in ICU. Furthermore, concern of safety outweighs potential benefits of physical activity for mechanically ventilated patients with ETT. This remains a prominent factor that impedes mobility in ICUs (Bakhru et al., 2015; Harrold et al., 2015; Morris & Herridge, 2007).

According to Cabana et al. (1999), in order to expedite the adoption of new guideline into clinical practice, knowledge is the first affected domain, followed by attitude, and lastly behaviour. Therefore, education on benefits, feasibilities and safety of EM is a key to change health caregivers’ perception towards EM implementation for ICU patients. Barriers to EM need to be identified, and facilitators of EM implementation need to be tailored in the local context, in order to enhance and sustain EM implementation in ICUs.

Remarkably, out‐of‐bed activities in the local context might be underestimated in this study. Certain hospitals in this study transferred ICU patients passively to bedside chairs and retained them in sitting position for two to three hours. The reason is to prevent orthostatic intolerance in an individual who has experienced prolonged bed rest. Signs of orthostatic intolerance begin to appear in 3 to 4 days of the beginning of bed rest, and it is preventable via regular exercise even during bed rest or regularly transfers patients from bed to chair (Topp et al., 2002). In MOH Malaysia EM protocol, passive transfer of patient from bed to chair is not included in any level of activities. However, in this study, we included patients who were passively transferred from bed to chair as level II activity.

### 5.2 Relationship between mode of ventilation and level of activity

Mode of ventilation, which included invasive (use of ETT and tracheostomy tube) and non‐invasive (use of non‐invasive positive pressure ventilation and venture mask/nasal prong) ventilation, was significantly associated with level of activity participated by ICUs patients in this study (*p* < .001). Similarly, Nydahl et al. (2014) also reported the proportion of patients mobilized out of bed differed significantly (*p* < .001) by type of airway used for ventilation, encompassed ETT (8%), tracheostomy (39%) and non‐invasive ventilation, NIV (59%). Furthermore, this study revealed that the invasive ventilated patient is at odds of 12.53 to stay in bed as compared to non‐invasive ventilated patient. The reasons could be more frequent use of deep sedation in mechanically ventilated patients via ETT (Nydahl et al., 2014), and the concern of risk associated with mobilizing ventilated patients outweighs the benefits (Jolley et al., 2014).

To optimize EM protocol implementation, standard equipment, such as walking frames, rehabilitation chairs, oxygen tank carriers and wheelchairs, appropriate or custom‐made facilities should be made available in ICUs in order. Safety of patients and healthcare providers could be ensured by having appropriate equipment while mobilizing ICU patients. This is because mobilizing a critically ill patient is a labour‐intensive task which requires appropriate facilities and skills. This was supported by Hodgson et al. (2013) who stated that despite having standard equipment, the safety of patients who underwent EM remained uncertain unless there were custom‐made facilities to accommodate all the necessary medical devices on patients during mobility session.

Early tracheostomy was proposed as one of the strategies to facilitate EM for ICU patients. The presence of ETT at day 14 (Bailey et al., 2007; Harrold et al., 2015; Thomsen et al., 2008) as compared to less than 7 days (Harrold et al., 2015) showed discrepancy in percentage of mobilization episodes with the presence of an ETT. The timing of tracheostomy insertion appeared to influence the frequency of EM activities in ICUs. Mobilization ICU patients while on mechanical ventilation via a tracheostomy occurred on more than one‐third of the possible occasions in the Scottish population (Harrold et al., 2015). Benefits of early tracheostomy reported in literature included decrease the days of mechanical ventilation and length of hospital stay and provide more opportunity of EM (Clum & Rumbak, 2007).

### 5.3 Adherence rate of EM protocol

Although the EM protocol was designed for MOH Malaysia ICUs since 2013, it is still a challenge to implement the evidence‐based recommendations into clinical practice. This was evidenced by the average adherence rate of 52.5% to EM protocol among the five MOH ICUs in Sarawak in this study. The gap between current practice and the evidence‐based knowledge seems to be a worldwide challenge including in the United States (Bakhru et al., 2015; Grol & Wensing, 2004) and Netherlands (Otterman et al., 2012). Therefore, bridging the gap between scientific knowledge and actual clinical care is crucial and thus has attracted most researchers’ attention in this decade (Dogherty et al., 2013; Weinert & Mann, 2008).

However, the average adherence rates of 71%–75% were reported in the MSIC annual report in the years 2015 and 2016 (Tai et al., 2017; Tong et al., 2016). The slightly lower adherence rate reported in this study was probably due to a more precise approach which was applied in calculating the adherence rate. In this study, the investigator included all the levels of activities achieved by patients in each shift. This was in contrast with the former reports mentioned, whereby only the lowest level of activity achieved by patients in day was chosen for adherence rate calculation (Tai et al., 2017; Tong et al., 2016). The investigator believed that the result of adherence rate would be more accurate by including all the levels of activities in the calculation as this reflects overall picture of EM practice.

### 5.4 Limitations

The findings on the prevalence and adherence rate of EM were based on the staff nurses’ documentation in the EM form. The results could be subjected to social desirability bias. People might over report the frequency of EM activities. Besides, this study only included five government ICUs in Sarawak. Therefore, the findings in this study were unable to represent the EM practice in Malaysia.

### 5.5 Recommendation for further research

Clinical study via observation is recommended in future surveys in order to accurately identify the prevalence of EM practice and adherence rate of EM protocol. A multiprofessional approach that uses both qualitative and quantitative data could be optimal for identifying barriers and potential strategies to overcome the problems that arise in implementing EM in ICU. Other states ICUs should be included into study to identify the current EM practice in Malaysia.

## 6 CONCLUSION

The conclusions derived from this study were as follows: (a) the prevalence of EM practice in the five government hospitals in Sarawak was 65.6% (*n* = 882 patient‐days); deep sedation and vasopressor infusion were the most reported barriers for avoidable and unavoidable factors of barriers to mobility, respectively; and patients receiving CRRT should no longer be precluded from EM on the basis that a vascular catheter or CRRT is in situ; (b) the average adherence rate of EM protocol for the five hospitals in Sarawak was 52.5%; and (c) result of Pearson chi‐square test showed there was a significant relationship between mode of ventilation and level of activity (*p* < .001). The invasive ventilated patient had 12.53 the odds to stay in bed as compared to non‐invasive ventilated patient. Barriers and facilitators of EM protocol adherence should be identified in the local context to foster EM implementation in ICUs.

## 7 RELEVANCE TO CLINICAL PRACTICE

This study provides insights into the current EM practice and the successfulness of EM protocol adoption in ICUs in the Sarawak context as it provides preliminary data on EM practice in the five government hospitals in Sarawak. The discrepancy level of mobility between Sarawak and other international countries was specifically highlighted in this study. This in turn may motivate multidisciplinary teams to maximize patients’ level of EM in the future.

Nevertheless, it is anticipated that high discrepancy of adherence rate among the hospitals will draw the clinicians’ attention to identify the problem and discuss on the quality improvement strategies to ascertain a better adherence towards the protocol. With this, patients’ duration on mechanical ventilation can be shortened, the length of hospitalization can be reduced, and patients’ functional status can be maximized upon hospital discharge. Therefore, the outcomes of ICU survivors can be improved, besides saving the cost of hospitalization. In addition, the study provides a more accurate estimation of adherence rate, which can be applied by the government agencies in their future annual reports.

### 7.1 What does this paper contribute to the wider global clinical community?

- Early mobility (EM) of patient during ICU stay is essential to enhance short‐term functional outcomes, maximize independent function and shorten duration of mechanical ventilation and hospital length of stay. Mode of ventilation should be taken into consideration in order to promote EM for ICU patients.
- Despite the feasibility and safety of EM, it remains a challenge for healthcare providers in implement the EM protocol for ICU patients. Barriers and facilitators of EM implementation should be identified from multidisciplinary team in order to enhance EM activities for ICU patients.

## CONFLICT OF INTEREST

The authors declare they have no competing interests.

## Supporting information

Supplementary Material

## Data Availability

The access link is http://ir.unimas.my/id/eprint/3125.
