# Supplementary material for: A cross‐sectional study of early mobility practice in intensive care units in Sarawak Hospitals, Malaysia
Source: Nurs Open. 2020 Oct 16;8(1):200–9. doi: 10.1002/nop2.619 (PMC7729545; doi:10.1002/nop2.619)
Supplement: Supplementary file 1 — Supplementary Material [file NOP2-8-200-s001.doc]

STROBE Statement—Checklist of items that should be included in reports of ***cross-sectional studies***

|  | Item No | Checklist items descriptions | Responded on page |
| --- | --- | --- | --- |
| **Title and abstract** | 1a | Indicate the study’s design with a commonly used term in the title or the abstract | 1 |
|  | 1b | Provide in the abstract an informative and balanced summary of what was done and what was found | 1 |
| Introduction | | |  |
| Background/rationale | 2 | Explain the scientific background and rationale for the investigation being reported | 2-4 |
| Objectives | 3 | State specific objectives, including any prespecified hypotheses | 5 |
| Methods | | |  |
| Study design | 4 | Present key elements of study design early in the paper | 5 |
| Setting | 5 | Describe the setting, locations, and relevant dates, including periods of recruitment, exposure, follow-up, and data collection | 5 |
| Participants | 6 | (*a*) Give the eligibility criteria, and the sources and methods of selection of participants | 6 |
| Variables | 7 | Clearly define all outcomes, exposures, predictors, potential confounders, and effect modifiers. Give diagnostic criteria, if applicable | 7 |
| Data sources/ measurement | 8 | For each variable of interest, give sources of data and details of methods of assessment (measurement). Describe comparability of assessment methods if there is more than one group | 9 |
| Bias | 9 | Describe any efforts to address potential sources of bias | × |
| Study size | 10 | Explain how the study size was arrived at | × |
| Quantitative variables | 11 | Explain how quantitative variables were handled in the analyses. If applicable, describe which groupings were chosen and why | 9 |
| Statistical methods | 12a | Describe all statistical methods, including those used to control for confounding (Descriptive analysis and Pearson correlation analysis and multiple stepwise regression analysis) | 9 |
|  | 12b | Describe any methods used to examine subgroups and interactions | × |
|  | 12c | Explain how missing data were addressed (Mean Imputation) | x |
|  | 12d | If applicable, describe analytical methods taking account of sampling strategy | × |
|  | 12e | Describe any sensitivity analyses | × |
| Results | | |  |
| Participants | 13a | Report numbers of individuals at each stage of study—eg numbers potentially eligible, examined for eligibility, confirmed eligible, included in the study, completing follow-up, and analysed | 10 |
|  | 13b | Give reasons for non-participation at each stage | × |
|  | 13c | Consider use of a flow diagram | × |
| Descriptive data | 14a | Give characteristics of study participants (eg demographic, clinical, social) and information on exposures and potential confounders | 11 |
|  | 14b | Indicate number of participants with missing data for each variable of interest | 11 |
| Outcome data | 15 | Report numbers of outcome events or summary measures | 11 |
| Main results | 16*a* | Give unadjusted estimates and, if applicable, confounder-adjusted estimates and their precision (eg, 95% confidence interval). Make clear which confounders were adjusted for and why they were included | 11 |
|  | 16*b* | Report category boundaries when continuous variables were categorized | × |
|  | 16*c* | If relevant, consider translating estimates of relative risk into absolute risk for a meaningful time period | × |
| Other analyses | 17 | Report other analyses done—eg analyses of subgroups and interactions, and sensitivity analyses | 12 |
| Discussion | | |  |
| Key results | 18 | Summarise key results with reference to study objectives | 13 |
| Limitations | 19 | Discuss limitations of the study, taking into account sources of potential bias or imprecision. Discuss both direction and magnitude of any potential bias | 18 |
| Interpretation | 20 | Give a cautious overall interpretation of results considering objectives, limitations, multiplicity of analyses, results from similar studies, and other relevant evidence | 13 |
| Generalisability | 21 | Discuss the generalisability (external validity) of the study results | x |
| Other information | | |  |
| Funding | 22 | Give the source of funding and the role of the funders for the present study and, if applicable, for the original study on which the present article is based | No funding |
